# The Surgical Diseases of Children

**Published:** 1887-03

**Authors:** 


The Surgical Diseases of Children. *By* Edmund Owen, M.D., F.R.C.S. Lea Brothers & Company, *Philadelphia, 1886. Octavo. Chicago:* A. C. McClurg & Company.

This little book forms one of a series of "Clinical Manuals for Practitioners and Students of Medicine." Each chapter is concise, pointed and admirably written. In mooted cases the author has chosen that theory of cause which furnishes the "best working basis" for successful treatment. Thus, he considers croup, diphtheria and membranous laryngitis practically identical, and early tracheotomy is strongly advocated.

*Tubage* is noticed briefly. With this operation Dr. Owen has evidently had no experience, but he pronounces it "an untrustworthy substitute for tracheotomy," and discountenances its use in children.

On acute intestinal obstruction he says, "Abdominal section is the only method of active treatment on which reliance can be placed, and its performance is demanded where the diagnosis has been made." And further, "Little trust should be placed in treatment by distention."

The illustrations comprise four chromo-lithographs and eighty-five engravings. M. E. B.

